# Supplementary material for: Multicomponent regulation of actin barbed end assembly by twinfilin, formin and capping protein
Source: Nat Commun. 2023 Jul 6;14:3981. doi: 10.1038/s41467-023-39655-3 (PMC10326068; doi:10.1038/s41467-023-39655-3)
Supplement: Supplementary file 3 — Description of Additional Supplementary Files [file 41467_2023_39655_MOESM3_ESM.pdf]

Description of Additional Supplementary Files

Supplementary Movie 1. : Elongation of formin-anchored filaments in presence of profilin and actin. Actin filaments were nucleated from coverslip-anchored formins by introducing a flow containing 1 µM G-actin (15% Alexa-488 labeled) and 0.5 µM profilin. The fluorescent filaments were then elongated in presence of 1 µM unlabeled G-actin and 4 µM profilin to ensure insertional elongation between fluorescent fragment and surface-anchored formins. These filaments were then exposed to a flow containing 0.2 µM unlabeled G-actin and 0.7 µM profilin (also see figure 1b).

Supplementary Movie 2. : Elongation of formin-anchored filaments in presence of CP, profilin and actin. Actin filaments were nucleated from coverslip-anchored formins by introducing a flow containing 1 µM G-actin (15% Alexa-488 labeled) and 0.5 µM profilin. The fluorescent filaments were then elongated in presence of 1 µM unlabeled G-actin and 4 µM profilin to ensure insertional elongation between fluorescent fragment and surface-anchored formins. These filaments were then exposed to a flow containing 0.2 µM unlabeled G-actin, 0.7 µM profilin and 50 nM CP (also see figure 1c).

Supplementary Movie 3. : Elongation of formin-anchored filaments in presence of CP, twinfilin, profilin and actin. Actin filaments were nucleated from coverslip-anchored formins by introducing a flow containing 1 µM G-actin (15% Alexa-488 labeled) and 0.5 µM profilin. The fluorescent filaments were then elongated in presence of 1 µM unlabeled G-actin and 4 µM profilin to ensure insertional elongation between fluorescent fragment and surface-anchored formins. These filaments were then exposed to a flow containing 0.2 µM unlabeled G-actin, 0.7 µM profilin, 50 nM CP and 1 µM mTwf1 (also see figure 1e).

Supplementary Movie 4. : Direct visualization of Formin-CP decision complex dynamics by single molecule imaging. Multicolor, merged, single molecule time-lapse movie of an actin filament (cyan) elongating with 649-mDia1 (magenta) bound to its barbed end in presence of 0.5 µM G-actin (15% Alexa-488 labeled, 0.5% biotin labeled) and 1 µM profilin and 10 nM 549-CP (yellow). The magenta arrowhead denotes the location of translocating formin, white arrowhead denotes the formation of the 549-CP:649-mDia1 decision complex and the yellow arrowhead marks the departure of 649-mDia1 from the decision complex, leaving 549-CP behind (also see figure 3a-c).
